# Paclitaxel Plus Platinum Neoadjuvant Chemotherapy Followed by Surgery *Versus* Primary Surgery in Locally Advanced Cervical Cancer—A Propensity Score Matching Analysis

**DOI:** 10.3389/fonc.2020.604308

**Published:** 2020-12-07

**Authors:** Yanan Zhang, Bin Li, Yating Wang, Shuanghuan Liu, Haibo Wang

**Affiliations:** ^1^ Department of Gynecology Oncology, National Cancer Center/National Clinical Research Center for Cancer/Cancer Hospital, Chinese Academy of Medical Sciences and Peking Union Medical College, Beijing, China; ^2^ Peking University Clinical Research Institute, Beijing, China

**Keywords:** uterine cervical neoplasms, locally advanced cervical cancer, neoadjuvant chemotherapy, primary surgery, propensity score matching

## Abstract

**Objective:**

To compare the efficacy and safety of neoadjuvant chemotherapy followed by surgery (NACTS) and primary surgery (PS) in locally advanced cervical cancer (LACC).

**Methods:**

LACC (stage IB2/IIA2, FIGO 2009) patients who accepted NACTS or PS in the Cancer Hospital of the Chinese Academy of Medical Sciences from 2007 to 2017 were enrolled, and a database was established. A 1:1 ratio propensity score matching (PSM) was performed for the NACTS group and PS group according to pretreatment characteristics. After PSM, the clinicopathological features and prognosis between the matched groups were compared.

**Results:**

Of 802 cases in the database, 639 met the inclusion criteria, with 428 received paclitaxel plus platinum NACTS, and 211 received PS. After PSM, the two groups had comparable pretreatment characteristics, with 190 cases in each group. In the NACTS group, the operation parameters were similar to the PS group except for the longer operation time (median 255 min *vs.* 239 min, *P* = 0.007); pathological intermediate-risk factors including tumor diameter (*P* < 0.001) and LVSI(+) (*P* < 0.001) were significantly decreased; fewer patients were with ≥2 intermediate-risk factors (10.5 *vs.* 53.2%, *P* < 0.001) so that the rate of adjuvant radiotherapy was reduced (54.2 *vs.* 70.0%, *P* = 0.002). DFS and OS were similar between the NACTS group and PS group (*P* > 0.05). However, for patients with tumor diameter ≥5 cm or SCC ≥5 ng/ml, DFS of the NACTS group was significantly prolonged (*P* = 0.016, *P* = 0.007).

**Conclusion:**

Paclitaxel plus platinum neoadjuvant chemotherapy can reduce adjuvant radiotherapy by decreasing pathological risk factors. Patients with tumor diameter ≥5 cm or SCC ≥5 ng/ml may obtain survival benefits.

## Introduction

Cervical cancer is the fourth most common malignant tumor in women, with approximately 570,000 new cases and 310,000 deaths worldwide in 2018 (1). Locally advanced cervical cancer (LACC) refers to patients of International Federation of Gynecology and Obstetrics (FIGO) stage IB2/IIA2. The survival of those patients is not satisfactory (2), and the treatment remains non-uniform: the National Cancer Network (NCCN) guidelines suggest concurrent chemoradiotherapy as the standard choice for LACC (category 1), and primary surgery is an alternative (category 2B) (3). Indeed, many medical centers perform primary surgery for LACC patients in Europe and Asia; especially 60% of Japanese medical centers perform primary surgery as reported (4). Given the bulky tumor of LACC, conducting primary surgery is usually difficult. And adjuvant therapy is often needed due to multiple pathologic risk factors that exist in LACC (5, 6).

Previous studies have shown that neoadjuvant chemotherapy could improve tumor resectability by reducing the tumor volume, avoid adjuvant radiation by reducing the pathological risk factors (7–11). But no consensus has been reached on whether NACTS can provide survival benefits (10, 12–21). Based on the previous efficacy evaluation, NCCN guidelines suggest paclitaxel plus cisplatin (TP) or paclitaxel plus carboplatin (TC) as the first-line therapy for recurrent and metastatic cervical cancer (3). However, few studies evaluated TP or TC neoadjuvant chemotherapy (10, 20). We aim to compare neoadjuvant chemotherapy of TP/TC regimen and primary surgery (PS) creatively. Besides, propensity score matching (PSM) was applied to balance the number and pretreatment characteristics between the NACTS group and PS group, which make the study similar to a randomized controlled trial.

## Patients and Methods

### Data Source and Collection

A database was established, containing patients with LACC who underwent surgery in the Cancer Hospital of the Chinese Academy of Medical Sciences from 2007 to 2017. Patients were divided into the NACTS group and PS group according to whether they accepted NACT or not. This study was conducted following the Declaration of Helsinki. The research protocol of this study was approved by the Ethics Committees of the National Cancer Center/Cancer Hospital at the Chinese Academy of Medical Sciences (No. NCC2016 ST-05).

### Treatment and Follow-Up

Pathologic diagnosis of cervical cancer was confirmed by preoperative biopsy, and the clinical stage was determined by two senior gynecologic oncologists through pelvic examinations. Enhanced CT scan and MRI were used to evaluate the parametrial invasion and the distant metastasis. All the patients underwent detailed pre-treatment examinations including blood routine, coagulation, biochemistry, electrocardiogram, and chest X-ray. Patients in the PS group accepted primary radical hysterectomy (Piver Type III or Q–M Type C) and pelvic lymph node dissection. Abdominal para-aortic lymph node dissection was performed when common iliac lymph nodes are positive or para-aortic lymph nodes are enlarged. The surgery approach included laparotomy and laparoscopy. According to the regression of the tumor, patients in the NACTS group accepted one to three cycles of neoadjuvant chemotherapy with TP/TC. The surgical procedure was the same as that of the PS group.

After surgery, patients with pathologic high-risk factors (lymph node metastasis, parametrial invasion, positive margin) received adjuvant concurrent chemoradiotherapy, while patients with two or more intermediate-risk factors (lymph-vascular space invasion, deep stromal invasion, and bulky tumors) or patients who met the Sedlis criteria (5) received radiotherapy or concurrent chemoradiotherapy. Patients were treated with three-dimensional conformal radiotherapy or by intensity-modulated radiotherapy. Fractions of 1.8 Gy were delivered up to five times a week, for twenty-five times overall. Patients with positive margin received brachytherapy. Patients with concurrent chemoradiotherapy received at least one cycle of weekly intravenous concomitant cisplatin (40 mg/m^2^) with a target of four or five cycles during external beam radiation (EBRT). Systemic chemotherapy was individualized according to the risk factors.

Follow-up procedures consisting of general and gynecologic examinations were performed every 3 months for 2 years and every 6 months thereafter. The follow-up period was to May 2019.

### Inclusion and Exclusion

Inclusion criteria included: (1) Patients with histopathologically proven squamous cell carcinoma, adenocarcinoma, or adenosquamous carcinoma of the uterine cervix; (2) newly diagnosed, previously untreated; (3) Eastern Cooperative Oncology Group (ECOG) performance score 0–1. Exclusion criteria included: (1) rare pathological types (neuroendocrine carcinoma or carcinosarcoma; (2) other neoadjuvant regimens rather than TP/TC; (3) a history of other malignancies; (4) incomplete medical records or lost to follow-up.

### Assessment of Response, Toxicity, Disease Free Survival, and Overall Survival

Tumor response of neoadjuvant chemotherapy was assessed by MRI or gynecologic examination; clinical response was evaluated according to the Response Evaluation Criteria in Solid Tumors guideline (RECIST) 1.1 (22).

Toxicity was graded referring to the National Cancer Institute Common Terminology Criteria for Adverse Events (NCI-CTCAE) 5.0 (23): grade III–IV myelosuppression is considered to be severe hematological toxicity.

Disease free survival (DFS) and overall survival (OS) were used to evaluate prognosis. DFS was defined as the interval from the date of surgery to the date of recurrence or the last follow-up. OS was defined as the interval from the date of surgery to the date of death or the last follow-up.

### Statistical Analysis

Propensity score matching was conducted based on the pretreatment risk factors, which included SCC (Squamous cell carcinoma antigen), stage, tumor diameter, surgical approach, and histological type. Logistic regression was used to estimate the propensity score. Propensity score matching was performed at a ratio of 1:1 with a caliper of 0.001.

SPSS 24.0 software (IBM SPSS., Chicago, IL) was used for data analysis. Continuous variables were compared by Student *t*-tests; categorical variables were analyzed using the Pearson Chi-square tests or Fisher’s exact tests. Survival analysis were conducted using the Kaplan–Meier method with log-rank tests. Univariable Cox regression analysis were used to identify risk factors of DFS. *P* < 0.05 was considered significant.

## Results

**Figure 1:**
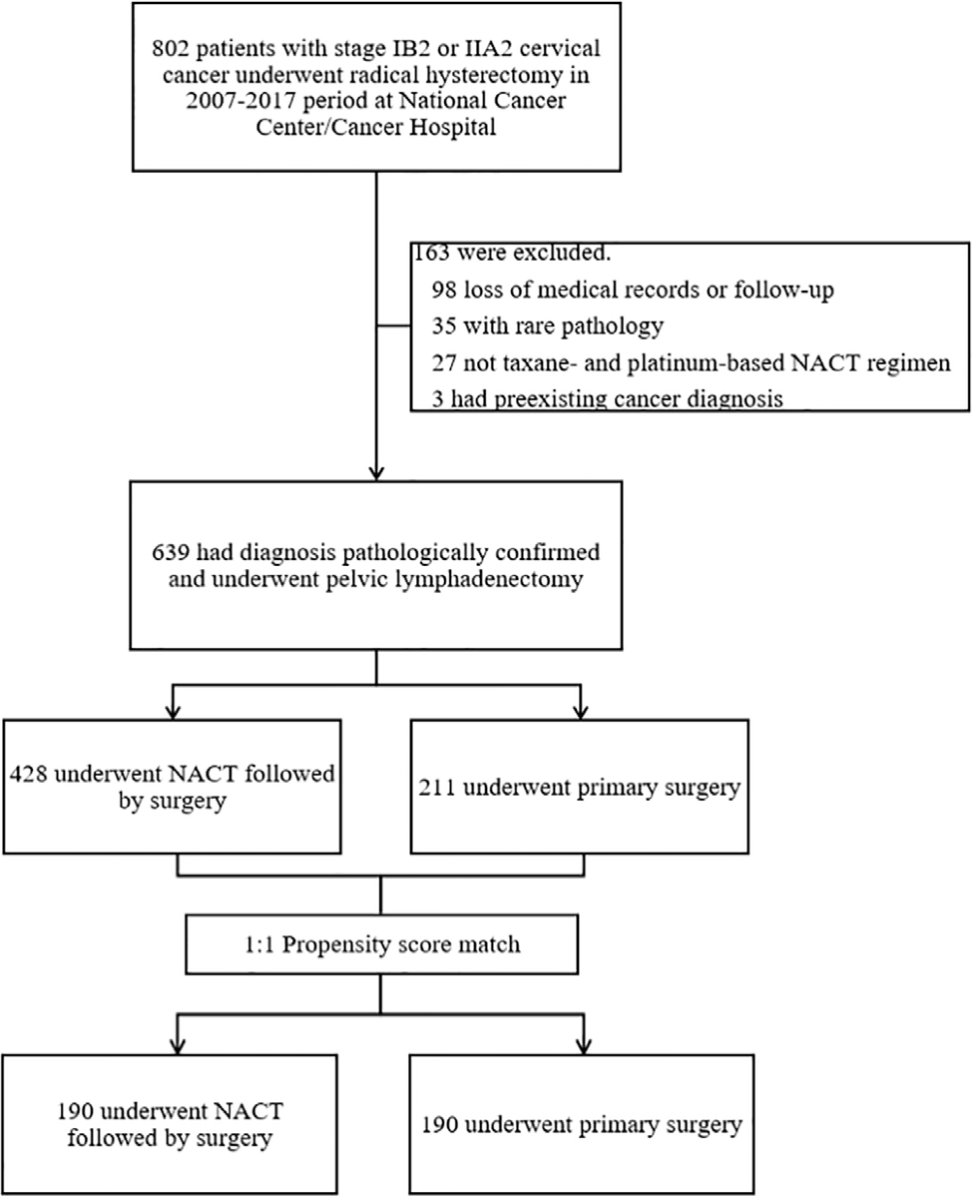
Flow chart of the patient selection process.

**Figure 1** shows the patient selection process. Of 802 patients in the LACC database, 639 patients who met the inclusion criteria were enrolled. There were 428 cases in the NACTS group and 211 cases in the PS group. Compared with the PS group, the NACTS group was with larger tumor, higher SCC value, more advanced stage, and a higher proportion of squamous cell carcinoma. After 1:1 propensity score matching, both groups included 190 cases with comparable pretreatment characteristics. Clinical and pathological characteristics including age, BMI (body mass index), tumor diameter, SCC value, stage, and pathological type are summarized in **Table 1**. We compared the efficacy and prognosis between the matched NACTS group and PS group.

**Table 1:** Characteristics between NACTS group and PS group before and after PSM.

Characteristics		Before PSM (n = 639)			After PSM (n = 380)		
		NACTS(n = 428)	PS(n = 211)	*p*	NACTS(n = 190)	PS(n = 190)	*p*
**Age (y)**		44.4 ± 7.9	43.9 ± 7.8	0.411	45.1 ± 7.1	43.9 ± 7.9	0.108
**BMI (kg/m^2^)**		24.1 ± 3.5	24.0 ± 3.3	0.700	24.3 ± 3.5	24.0 ± 3.3	0.286
**Diameter (cm)**	4-5	184(43.0%)	131(62.1%)	<0.001	115(60.5%)	115(60.5%)	1.000
	5-6	147(34.3%)	62(29.4%)		59(31.1%)	59(31.1%)	
	≥6	97(22.7%)	18(8.5%)		16(8.4%)	16(8.4%)	
**SCC (ng/ml)**	<1.5	96(22.4%)	75(35.6%)	<0.001	67(35.3%)	66(34.7%)	0.993
	1.5-5	147(34.4%)	73(34.6%)		70(36. 8%)	69(36.3%)	
	5-10	89(20.8%)	26(12.3%)		26(13.7%)	26(13.7%)	
	≥10	90(21.0%)	29(13.7%)		27(14.2%)	29(15.3%)	
	unknown	6(1.4%)	8(3.8%)		–	–	
**Stage**	IB2	316(73.8%)	177(83.9%)	0.004	162(85.3%)	163(85.8%)	0.884
	IIA2	112(26.2%)	34(16.1%)		28(14.7%)	27(14.2%)	
**Pathology**	SC	415(97.0%)	196(92.9%)	0.026	189(99.5%)	189(99.5%)	1.000*
	AC	10(2.3%)	14(6.6%)		1(0.5%)	1(0.5%)	
	ASC	3(0.7%)	1(0.5%)		–	–	
**Surgery**	ARH	384(89.7%)	194(91.9%)	0.368	178(93.7%)	179(94.2%)	0.830
	LRH	44(10.3%)	17(8.1%)		12(6.3%)	11(5.8%)	

SC, squamous carcinoma; AC, adenocarcinoma; ASC, adenosquamous carcinoma, ARH, abdominal radical hysterectomy; LRH, laparoscopic radical hysterectomy.

*Fisher’s exact test.

Concerning neoadjuvant chemotherapy response in the NACTS group, there were 25 (13.2%) cases of complete response (CR), 130 (68.4%) cases of partial response (PR), 31 cases of stable disease (SD), 4 cases of progression disease (PD). Clinical responders (CR + PR) accounted for 81.6%. Eight cases (4.2%) suffered grade III/IV leukopenia, and no one experienced grade III/IV thrombocytopenia or anemia.

The operation time of the NACTS group was increased (255.1 ± 58.1 *vs.* 239.6 ± 52.5 min, *P* = 007), but the other operation parameters including surgical complications were similar in the NACTS and PS group. As shown in **Table 2**.

**Table 2:** Surgery parameters between NACTS group and PS group.

	NACTS (n = 190)	PS (n = 190)	
**Complications**			
** Ureteral injury**	0	2	
** Bladder injury**	0	1	
** Ileus**	1	2	
** Abdomen wound dehiscence**	3	3	
**Surgery parameters**			
** Transfusion rate (%)**	75 (39.5%)	66 (34.7%)	*P* = 0.339
** Blood loss, ml**	403.3 ± 253.8	395.5 ± 261.7	*P* = 0.767
** Operate time, min**	255.1 ± 58.1	239.6 ± 52.5	*P* = 0.007
** Catheter retention, d**	14.0 ± 8.3	15.1 ± 8.4	*P* = 0.216
** Hospital stay, d**	14.7 ± 6.7	15.7 ± 6.2	*P* = 0.122

The incidence of high-risk factors including lymph node metastasis, parametrium invasion, and positive margin was similar between the NACTS and PS groups (*P* > 0.05), while intermediate-risk factors including LVSI(+) and tumor diameter were significantly different between the two groups. LVSI(+) was significantly lower in the NACTS group (14.2 *vs.*31.6%, *P* = 0.001). LACC patients in the PS group were all with bulky tumors (>4 cm), but 73.2% of patients in the NACTS group were with tumors <4 cm. There were fewer patients with ≥2 intermediate-risk factors in the NACTS group (10.5 *vs.* 53.2%, *P* = 0.001), and 21.6% had no pathological risk factors after surgery. Fewer patients received adjuvant radiotherapy (including concurrent chemoradiotherapy) in the NACTS group (54.2 *vs.* 70.0%, *P* = 0.002) as shown in **Table 3**.

**Table 3:** Pathologic characteristics and adjuvant therapy between NACTS group and PS group.

		NACTS (n = 190)	PS (n = 190)	*P*
**Lymph node number**	–	34.4 ± 12.1	33.0 ± 11.1	0.233
**Lymph node metastasis**	Yes	41(21.6%)	50(26.3%)	0.279
	No	149(78.4%)	140(73.7%)	
**Parametrium invasion**	Yes	2(1.1%)	0(0%)	0.499*
	No	188(98.9%)	190(100%)	
**Positive margin**	Positive	1(0.5%)	0(0%)	1.000*
	Negative	189(99.5%)	190(100%)	
**LVSI**	Positive	27(14.2%)	60(31.6%)	<0.001
	Negative	163(85.8%)	130(68.4%)	
**Deep stromal invasion**	Yes	141(74.2%)	130(68.4%)	0.212
	No	49(25.8%)	60(31.6%)	
**Tumor diameter**	<4cm	139(73.2%)	0(0%)	<0.001
	≥4cm	51(26.8%)	190(10 0%)	
**Risk factor**	Yes	149(78.4%)	190(100.0%)	<0.001
	None	41(21.6%)	0(0%)	
**≥2 intermediate-** **risk factors**	Yes	20(10.5%)	101(53.2%)	<0.001
	No	170(89.5%)	89(46.8%)	
**≥1 high-risk factor**	Yes	43(22.6%)	50(26.3%)	0.404
	No	147(77.4%)	140(73.7%)	
**Adjuvant therapy**	Yes	129(67.9%)	144(75.8%)	0.087
	None	61(32.1%)	46(24.2%)	
**Radiation (Radiation/** **Chemoradiation)**	Yes	103(54.2%)	133(70.0%)	0.002
	No	87(45.8%)	57(30.0%)	
**Systemic Chemotherapy**	Yes	26(13.7%)	11(5.8%)	0.336
	No	164(86.3%)	179(94.2%)	

*Fisher’s Exact Test.

**Figure 2:**
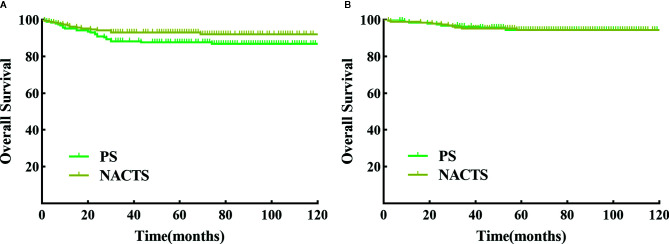
DFS and OS between NACTS group and PS group. **(A)** Survival curve of DFS between NACTS and PS (*P* = 0.098). **(B)** Survival curve of OS between NACTS and PS (*P* = 0.963).

The follow-up period ranged from 1 to 126 months, with an average of 70 months. There were 14 relapsed cases in the NACTS group, including seven local recurrences (50%) and seven distant recurrences; there were 24 relapsed cases in the PS group, with eight local recurrences (33.3%) and 16 distant recurrences (66.7%). The recurrence pattern (local or distant recurrence) was similar between the two groups (*P* = 0.311). DFS (*P* = 0.098) and OS (*P* = 0.963) were comparable in the NACTS group and PS group. The 5-year DFS of the two groups was 93.2 and 87.7%, respectively, and the 5-year OS was 94.5% in both groups. Survival curves are shown in **Figures 2A, B**. Moreover, the 5-year DFS and 5-year OS of the clinical responders in the NACTS group were 94.2 and 95.8%, which were not significantly better than those in the PS group (*P* = 0.061, *P* = 0.587).

**Figure 3:**
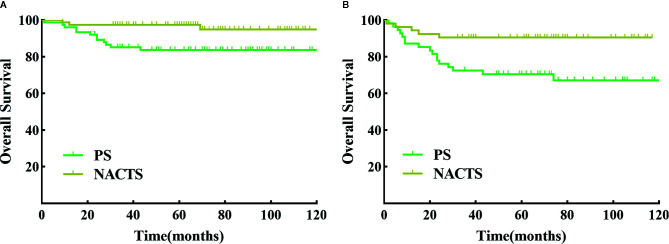
DFS between NACTS and PS in stratification. **(A)** Survival curve of DFS between NACTS and PS in patients with tumor diameter ≥5 cm (*P* = 0.016). **(B)** Survival curve of DFS between NACTS and PS in patients with SCC ≥5 ng/ml (*P* = 0.007).

We further conducted a stratified survival analysis by stage, tumor diameter, and SCC value. Survival in NACTS and PS groups was not different according to IB2 or IIA2 stage stratification analysis (*P >* 0.05). In patients with tumor diameter ≥5 cm, the NACTS group showed longer DFS (*P* = 0.016) but a similar OS (*P* = 0.633) compared to the PS group, with 5-year DFS of 94.8 and 83.7% respectively. In patients with SCC value ≥5 ng/ml, the NACTS group showed longer DFS (*P* = 0.007) but a similar OS (*P*=0.423) compared to PS group, with 5-year DFS of 90.6 and 70.5% respectively. However, in patients with tumor diameter <5 cm or SCC <5 ng/ml, DFS and OS were comparable in the NACTS group and PS group (*P >* 0.05). The DFS survival curves of patients with tumor diameter ≥5 cm and patients with SCC value ≥5 ng/ml were shown in **Figures 3A, B**.

**Figure 4:**
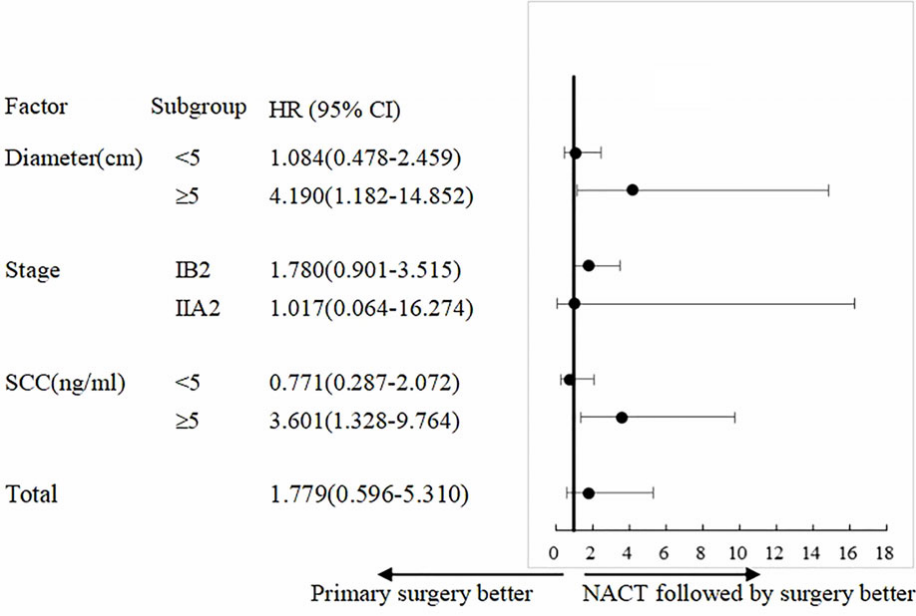
Subgroup analysis of PFS. Treatment effect with NACTS compared with PS in several subgroups.

COX univariate analysis showed that in patients with tumor diameter ≥5 cm, the PS group had 4.19-fold higher risk of recurrence than that of the NACTS group (95%CI 1.182–14.852, *P* = 0.026); in patients with SCC ≥5 ng/ml, the PS group had 3.60-fold higher risk of recurrence than that of the NACTS group (95%CI 1.328–9.764, *P*=0.012). The analysis of recurrence risk is shown in **Figure 4**.

## Discussion

The present research evaluates NACT in locally advanced cervical cancer using the statistical method of propensity score matching for the first time. Propensity score matching can balance confounding factors between each group, eliminate treatment selection bias, and ensure that the groups are comparable, which make the study similar to a randomized controlled trial (24). With this statistical method, we selected the NACTS group (n = 190) and PS group (n = 190) from all 802 LACC patients treated in our center in the past 10 years. The two groups had equal case numbers and balanced pretreatment factors, which makes the results of this study more objective and accurate. This is also the largest single-center study to date.

In this study, the NACTS group adopted the TP/TC regimen, the proportion of clinical responders (CR + PR) was 81.6%, and the incidence of severe leukopenia was only 4.2%. Gupta et al. (25) reported that the clinical response rate was 78.5% with TC neoadjuvant chemotherapy, while Cho et al. (10) discovered 83.3% achieved clinical response with TC/TP regimen. Only 0–8.9% severe leukopenia was observed in neoadjuvant chemotherapy with TP regimen (21, 26, 27). The results of these studies are similar to our study. However, patients who accepted BOMP (cisplatin, vincristine, bleomycin, mitomycin) regimen in the phase III clinical trial―JCOG0102 showed 66% overall response, 41% severe leukopenia, and 27% severe thrombocytopenia (18). In a recent phase II clinical trial, Mori et al. (28) evaluated the efficacy of irinotecan combined with nedaplatin in neoadjuvant chemotherapy: with 81.2% overall clinical response, the incidence of severe leukopenia was as high as 40.7%. Therefore, TP/TC regimen is with high response and rare adverse reaction, which is superior to other regimens for neoadjuvant chemotherapy.

The effect of neoadjuvant chemotherapy on surgical morbidity has been a concern. We found the average operation time in NACTS group was 15 minutes longer than that in the PS group. Zhao et al. (20) also found 24 minutes longer in the NACTS group. The above results indicate that neoadjuvant chemotherapy reduces the tumor size but still increases the difficulty of operation. In this regard, several studies have suggested that chemotherapy-induced fibrosis and calcification will increase the difficulty of tissue separation; the operator should fully estimate (10, 29, 30). However, we found similar operation parameters in the two groups including hospital stay, blood loss, and surgery complications, indicating that neoadjuvant chemotherapy has no impact on the safety of operation, which is supported by other studies (15, 16, 18, 20, 31).

We believe that neoadjuvant chemotherapy can reduce adjuvant therapy through decreasing pathological risk factors. However, these studies have not reached a consensus on which risk factors have been reduced by neoadjuvant chemotherapy, nor have they analyzed according to high-risk factors or intermediate-risk factors (10, 12, 13, 16, 18). A meta-analysis found that NACTS reduced the rate of adjuvant radiotherapy in LACC by reducing a high-risk factor―lymph node metastasis (7). However, there was no difference in the rate of lymph node metastasis between the NACTS group and PS group in our study, which is supported by Yan et al. (21) and Hu et al. (17), both of whom found that NACTS could not reduce lymph node metastasis. We found that the intermediate-risk factors――bulky tumor and LVSI(+), were significantly decreased in the NACTS group, which is consistent with other studies (10, 12, 17). FIGO guidelines recommend adjuvant radiotherapy for patients with more than two intermediate-risk factors (32). Therefore, this study suggests that NACTS can reduce adjuvant radiotherapy by reducing these intermediate-risk factors. Complication of adjuvant radiotherapy is the main factor affecting the life quality, and reducing the use of adjuvant radiotherapy is the most significant value of NACTS. Besides, 21.6% of patients in the NACTS group had no pathological risk factors, including 13.2% with no residual tumor. There is no consensus on whether such patients need adjuvant therapy. At present, two studies agree that LACC patients without residual tumor or residual tumor with <3 mm stromal invasion after NACTS need no adjuvant therapy (8, 33). However, these two studies are controversial for patients with residual tumor >3 mm stromal invasion. Sun et al. (33) believe that such patients cannot benefit from adjuvant therapy, while Landoni et al. (8) think adjuvant chemotherapy is a benefit for such patients rather than radiotherapy.

It is crucial to find out whether NACTS can improve the prognosis of LACC, but there is still great controversy (10, 12–21). Retrospective studies have certain problems such as unbalanced baseline characteristics and non-uniform chemotherapy regimens, so it is difficult to draw objective conclusions (17, 20, 21). Prospective research was carried out more than ten years ago with old chemotherapy regimens and controversial inclusion criteria (including patients with stage IIB) (12, 13, 15, 16, 18). But we evaluate NACTS with paclitaxel plus platinum or carboplatin regimen—the first-line regimen recommended by NCCN guidelines for recurrent and metastatic cervical cancer. Although this is a retrospective study, the number of cases was large, and the pretreatment characteristics of the two groups were matched and balanced by propensity score matching. Nevertheless, we did not found that NACTS improved the prognosis, and even those clinical responders of NACT did not show a survival benefit. But we don’t deny the value of NACTS in improving survival. After further stratification analysis, we found that NACTS significantly increased the DFS of patients with tumor diameter ≥5 cm. In this regard, Sardi et al. (12) and Cai et al. (13) suggested that NACTS can improve the prognosis of LACC with bulky tumors by increasing the resection rate. Huang et al. suggest that tumor diameter >5 cm is an independent risk factor for survival in LACC patients treated with NACTS (34). Therefore, it is considered to take tumor diameter >5 cm as an indicator for NACTS. Also, it is reported that SCC is a risk factor for the prognosis of LACC (35–37). Taking 5ng/ml as the cut-off value, SCC can effectively predict the response of NACT and prognosis (38, 39). We conducted a stratified analysis of SCC <5 ng/ml and SCC ≥5 ng/ml and found that patients with SCC ≥5 ng/ml can benefit from NACTS, who showed significantly longer DFS. SCC ≥5 ng/ml as another indication for NACTS is worth exploring.

Although we performed propensity score matching in this study, it is still a retrospective study. A prospective study is warranted to evaluate the efficacy, especially survival benefits of paclitaxel and platinum neoadjuvant chemotherapy for LACC.

## Conclusion

Paclitaxel and platinum-based neoadjuvant chemotherapy is safe and effective, which can reduce adjuvant radiotherapy by decreasing postoperative risk factors in LACC. Patients with tumor diameter ≥5 cm or SCC ≥5 ng/ml may obtain survival benefits.

## Data Availability Statement

The raw data supporting the conclusions of this article will be made available by the authors, without undue reservation.

## Ethics Statement

The studies involving human participants were reviewed and approved by the Ethics Committees of the National Cancer Center/Cancer Hospital at the Chinese Academy of Medical Sciences. The patients/participants provided their written informed consent to participate in this study.

## Author Contributions

YZ took part in the conceptualization, data curation, and writing and preparation of the original draft. BL took part in the conceptualization, project administration, and writing, reviewing, and editing the manuscript. YW took part in the formal analysis, investigation, and data curation. SL took part in the formal analysis, investigation, and data curation. HW was in charge of the software and formal analysis. All authors contributed to the article and approved the submitted version.

## Funding

This work was supported by the Research and Achievement Promotion of the Characteristic Clinical Practice in Capital (Z171100001017115).

## Conflict of Interest

The authors declare that the research was conducted in the absence of any commercial or financial relationships that could be construed as a potential conflict of interest.
